# Association between peak inspiratory flow rate and hand grip muscle strength in hospitalized patients with acute exacerbation of chronic obstructive pulmonary disease

**DOI:** 10.1371/journal.pone.0227737

**Published:** 2020-01-31

**Authors:** Arash Samarghandi, Octavian C. Ioachimescu, Rehan Qayyum

**Affiliations:** 1 Division of Pulmonary, Allergy, Emory University School of Medicine, Critical Care and Sleep Medicine, Atlanta, Georgia; 2 Division of Hospital Medicine, Virginia Commonwealth University School of Medicine, Richmond, Virginia, United States of America; University of Rouen-Normandie, FRANCE

## Abstract

**Rationale:**

Ineffective peak inspiratory flow rate (PIFR) generation in patients using inhalers results in insufficient drug delivery to the lungs and poor clinical outcomes. Low inspiratory muscle strength is associated with suboptimal PIFR.

**Objective:**

To examine in a prospective study the relationship between PIFR and skeletal muscle strength using hand grip strength (HGS) as a surrogate.

**Methods:**

Adult patients admitted with acute exacerbation of chronic obstructive pulmonary disease (COPD) were enrolled. PIFR was measured within 48 hours before discharge. PIFR below 60L/min was considered suboptimal. HGS was measured using a handheld dynamometer. Any readmissions and emergency department visit data were collected. The associations between PIFR, HGS, 30 and 90-day COPD and all-cause readmissions were examined, without and with adjustment for age, race and gender.

**Results:**

Of the 75 enrolled patients, 56% had suboptimal PIFR; they were older (63.9±9.7 vs. 58.2±7.7 years) and had significantly lower HGS (24.2±11.1 vs. 30.9±10.9 Kg) compared to those with optimal PIFR. There were no significant differences between the two PIFR groups by gender, race, history of coronary artery disease, congestive heart failure, hypertension or functional scores. Each kilogram increase in HGS was associated with 0.50 (95%CI 0.18–0.89, p = 0.003) L/min increase in PIFR. We did not observe an association between PIFR and 30 or 90-day readmission rates.

**Conclusion:**

We found a significant association between HGS and PIFR in hospitalized patients with acute exacerbations of COPD. Whether interventions aimed at increasing skeletal muscle strength also result in improvement in PIFR remains unclear and need further study.

## Introduction

Patients with chronic obstructive pulmonary disease (COPD) are high users of healthcare. The estimated annual cost of COPD management in the US has been estimated at up to 50 billion dollars, a third of which is directly related to inpatient hospitalization for COPD exacerbations[1]. About one in five patients admitted with an acute exacerbation of COPD (AECOPD) is re-hospitalized within 30 days of hospital discharge.[2,3] Inhaled drugs are the cornerstone for prevention of COPD exacerbation and hospitalization[4], and provide better pulmonary bioavailability, lower dose requirement and less systemic toxicities than the oral or injectable drugs. Current recommendations advise that all patients hospitalized with AECOPD should be discharged on a controller inhaler to reduce risk of exacerbation and to improve control of COPD.[5,6]

Effective peak inspiratory flow rate (PIFR) generation is associated with optimal drug delivery to distal airways and lung parenchyma, and better clinical outcomes in those on inhaler therapy, especially dry powder inhaler (DPI) users.[7,8,9] Several studies have investigated the effect of suboptimal PIFR on the efficacy of different DPIs in preventing COPD exacerbations, however, most trials have been done in outpatient settings and in patients with controlled COPD.[10,11,12] In one study, subjects with suboptimal PIFR were found to have fewer days to next COPD exacerbation compared to patients with optimal PIFR.[13]

One determinant of PIFR is the strength of inspiratory muscles.[14] COPD is associated with skeletal respiratory muscle dysfunction and reduced muscle endurance.[15–17]^.^ Limb muscle dysfunction has been well described in COPD. For example, the collaborative guidelines issued in 2014 by American Thoracic Society and European Respiratory Society discuss limb atrophy and weakness in stable COPD, as well as significant skeletal muscle dysfunction from the baseline during acute exacerbation of COPD.[18] However, the relationship between PIFR and skeletal muscle strength in hospitalized patients has not been examined. It is important to know whether patients with sub-optimal PIFR have impaired skeletal muscle strength as well, which may correlate with a higher risk of frailty. Our objective was to prospectively examine the relationship between PIFR and hand muscle strength in hospitalized patients with AECOPD.

## Methods

Hospitalized adult patients who were admitted with AECOPD at Virginia Commonwealth University Hospital (VCU) from January 2018 to June 2018 were eligible for enrollment in this prospective study. We excluded patients with tracheostomy and those with physical or cognitive limitations. Patients were approached for written consent if their treating clinician expected them to be discharged within the next 48 hours. Demographic and clinical information data were obtained from medical records. The study was approved under Virginia Commonwealth University School of Medicine (VCU) Institutional Review Board and ethics committee under expedited review (IRB HM20012033)

PIFR was measured by using In-Check^®^ DIAL device (Alliance Tech Medical) at bedside. In-Check® DIAL device is a small simple dial, that has been used in previous studies to measure PIFR.[10,12,19]

The device is well validated,[20,21] capable of measuring inspiratory flow rates between 15 and 120 L/min. The accuracy is within ±10% or within 10 L/min, whichever is greater.[22] This device has the option to be set for a specific level of resistance to airflow. We set the resistance to simulate the Discus^®^ DPI; Discus^®^ DPI is a commonly prescribed DPI, with low to medium resistance.[23] Patients were instructed to inhale as fast as possible after a complete exhalation in a sitting position. Patients were allowed to attempt up to 3 times, with 1–3 minutes breaks between attempts; the breaks were allowed so that patients can return to their baseline respiration ability. We used the mean of the three attempts in our analysis. In accordance with prior literature, a PIFR of >60 L/min was considered optimal force for drug delivery of a DPI, while a PIFR below 60L/min was considered suboptimal.[24–26]

Muscle strength was measured with hand grip strength (HGS) using a handheld dynamometer, Jama®, in kilograms.[27] The test was performed at bedside with the subject also in sitting position, shoulder adducted, and elbow on the side of the dominant hand flexed to 90 degrees. Subject was asked to squeeze the dynamometer handle as hard as possible for 3–5 seconds. The test was performed with two attempts, with one-minute breaks in-between trials, and the mean of the two was used in data analyses.

The level of functional limitation due to dyspnea and COPD related symptoms were assessed via modified Medical Research Council (mMRC) dyspnea scale and COPD Assessment Test (CAT) for enrolled patients. The mMRC is a 5-point (0–4) scale based on the severity of dyspnea, “0” being minimal dyspnea with activity and “4” being dyspnea at rest. The CAT includes eight items related to the severity of cough, sputum, dyspnea, chest tightness, capacity for exercise and activities, confidence, sleep quality and energy levels, the score ranging from 0 to 40.[28]

Subjects were also asked about the contact phone number where they could be reached at 30 and 90 days after discharge. Rehospitalization data after index hospital admission were collected via chart review at 30 and 90 days. Those with no readmission on chart review at day 30, were contacted via phone and inquired about any emergency department visit or hospitalization at any other facilities; same process was done at 90 days after index admission. Those with 30-day readmission due to any cause were excluded from the 90-day follow-up.

Data were summarized using mean ± standard deviation for continuous variables and with frequencies for categorical variables. Difference between the groups were measured using independent t-test, rank sum test, or chi-square test as appropriate. Linear regression models were used to assess the relationship between PIFR and HGS, and logistic regression models were used to assess the relationship between dichotomized PIFR and HGS, without and with adjustment for age, gender, and race. To examine the relationship between readmissions at 30 or 90 days and PIFR, we used logistic regression models, without and with adjustment for age, gender, race, and mMRC score. All regression models were performed using bootstrap with 1000 replications using Stata 14.1 for Windows (College Station, Texas, USA).

## Results

Of 75 patients enrolled in the study, 56% (n = 42) had sub-optimal PIFR (S-PIFR). The mean S-PIFR was 46.1±10 vs 74.2±11 L/min,(p = 0.76) in optimal PIFR (O-PIFR) group. Patients with S-PIFR were older (63.9±9.7 years vs 58.2±7.7 years, p = 0.008), as compared to those with O-PIFR. Sixty percent of patients with S-PIFR were current smokers. The mean CAT score was 25±8.3 for S-PIFR cohort and 26.3±6.8 for O-PIFR (p = 0.4). Mean mMRC was 2.9±0.8 in S-PIFR group vs 2.6±1.0 for O-PIFR (p = 0.13). There was no statistically significant difference in CAT and mMRC between the two cohorts. There were also no significant differences by gender, race, history of coronary artery disease, congestive heart failure, or hypertension between the two PIFR groups (Table 1). The numbers of patients with concomitant asthma was higher in the O-PIFR than in S-PIFR group (12/18 (66.6%) vs 6/18 subjects (33.3%), p = 0.03).

**Table 1:** Patient demographics and clinical characteristics for all participants and by peak inspiratory flow rate (PIFR) categories suboptimal PIFR (S-PIFR) and optimal PIFR (O-PIFR). *P* values: S-PIFR vs O-PIFR.

	*All*	*S-PIFR*	*O-PIFR*	*p value*
***Patients, n (%)***	75 (100)	42 (56)	33 (44)	
***Age, mean (SD)***	61.4 ±9.2	63.9 ±9.7	58.2 ±7.7	**0.008**
***Female, n (%)***	42 (56)	26 (61.2)	16 (48.4)	0.34
***Race***				
***White, n (%)***	21 (28)	11 (26.1)	10 (30.3)	0.79
***Other, n (%)***	54 (72)	31 (73.8)	23 (69.7)	
***Current smoker, n (%)***	46 (61.3)	25 (59.5)	21 (63.6)	0.81
***CAT score, mean (SD)***	25.6 ±7.6	25.0 ±8.3	26.3 ±6.8	0.44
***mMRC, mean (SD)***	2.7 ±0.9	2.9 ±0.8	2.6 ±1.0	0.13
***Coronary artery disease, n (%)***	16 (31.3)	9 (21.4)	7 (21.2)	0.99
***Hypertension, n (%)***	51 (68)	32 (76.1)	19 (57.5)	0.13
***Diabetes mellitus, n (%)***	26 (34.6)	14 (33.3)	12 (36.3)	0.81
***Congestive heart failure, n (%)***	20 (26.6)	13 (30.9)	7 (21.2)	0.43
***Asthma, n (%)***	18 (24)	6 (33.3)	12 (66.6)	**0.03**
***Hand Grip Strength (kg), mean (SD)***	27.2 ±11.4	24.2 ±11.1	30.9 ±10.9	**0.003**
***PIFR (L/min), mean (SD)***	58.6 ±17.6	46.1 ±10.4	74.2 ±11.4	0.76

### Association between Hand Grip (HGS) and PIFR

**Fig 1:**
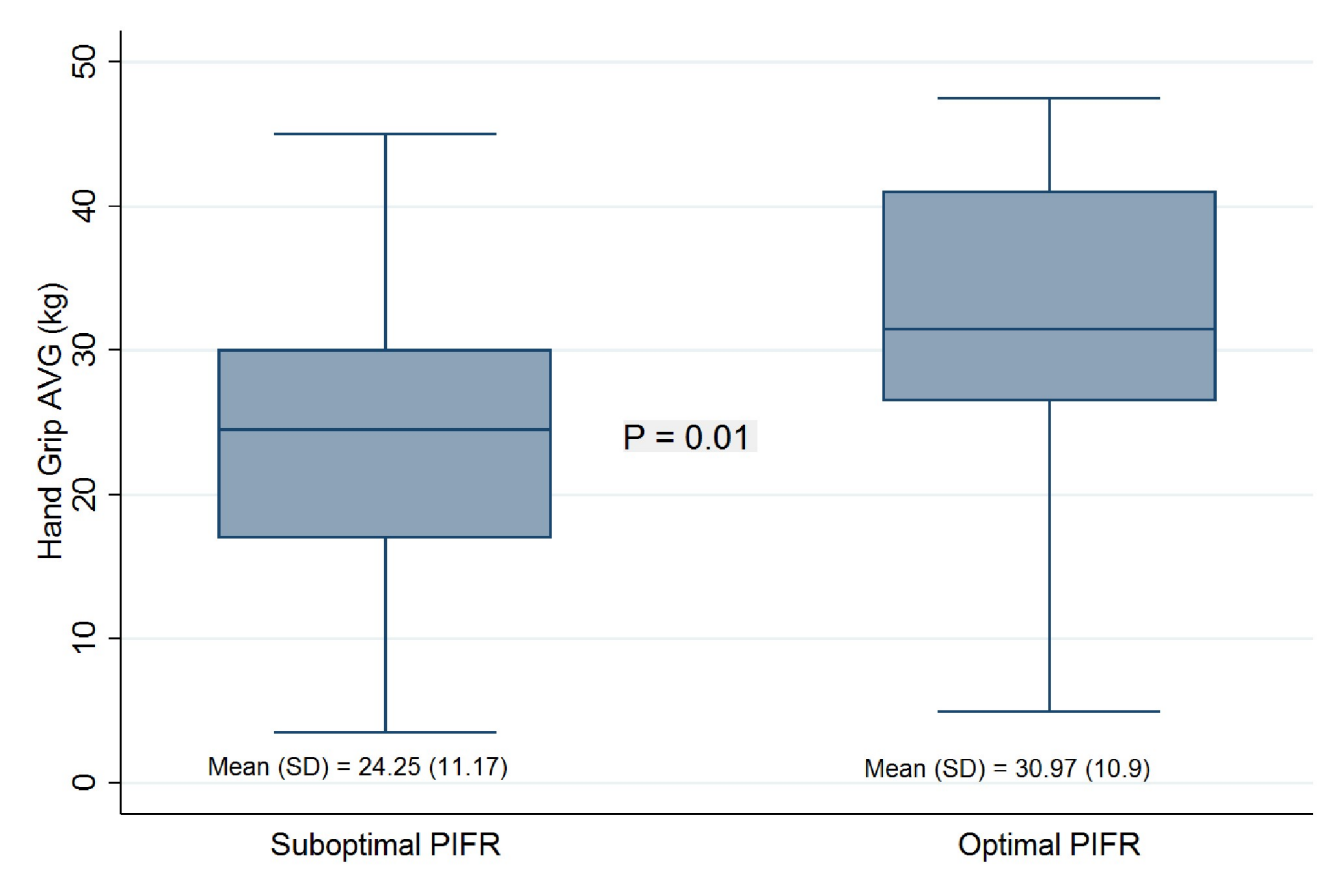
Association between peak inspiratory flow rate (PIFR) and Hand grip strength (HGS). The middle line in the box is the median HGS, the upper and lower boundaries of the box represent 75^th^ and 25^th^ percentiles of HGS distribution, the whiskers are 1.5*IQR from 75^th^ and 25^th^ percentiles from HGS.

**Fig 2:**
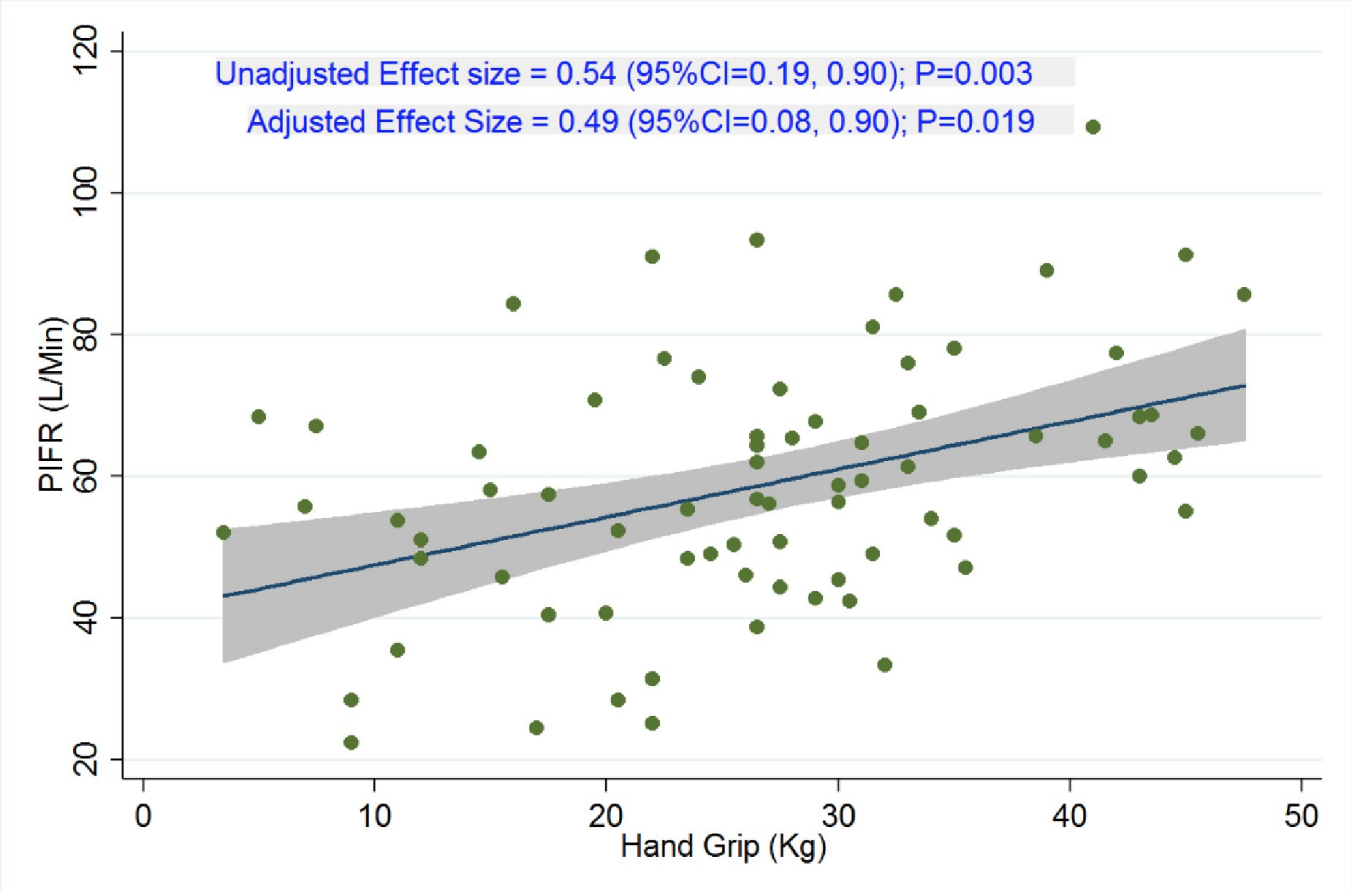
Association between peak Inspiratory flow rate (PIER, L/min) and Hand Grip Strength(HGS, kg) as continuous variables.

The mean hand grip strength (HGS) was 27.2±11.4 kg in the study cohort. Patients in the S-PIFR group had significantly lower HGS than those in the O-PIFR group (24.2±11.1 vs 30.9±10.9 kg, p = 0.01, Figs 1 & 2). Each kilogram increase in HGS was associated with 0.54 L/min (95% confidence interval [CI], 0.18–0.89; *P =* 0.003) increase in PIFR in unadjusted model. The association remained significant when adjusted for age, sex, and race (effect size = 0.49; 95%CI = 0.08 to 0.90; P = 0.02).

### Association of PIFR with readmission rate

The 30-day all-cause readmission rate was 21% (16/75), while the 90-day all-cause readmission rate was 37% (28/75). While our data did not show a statistically association between PIFR and 30 or 90-day readmission rates, patients in the S-PIRF group had a trend towards higher rate of 30-day (11/42 vs 5/33 patients, RR 0.36 (0.06–2.3), p = 0.2) and 90-day all-cause readmission rate (17/42 vs 11//33, RR 0.71 (0.20–2.5, p = 0.6) as compared to the O-PIFR group (Table 2).

**Table 2:** Readmission rates for all patients, suboptimal PIFR (S-PIFR) and optimal PIFR (O-PIFR). CI: confidence interval; COPD: chronic obstructive pulmonary disease; OR: Odds Ratio, *P* values: S-PIFR vs O-PIFR.

	*Total (n = 75)*	*S-PIFR (n = 42; 56%)*	*O-PIFR (n = 33; 44%)*	*OR (95%CI)*	*p value*
***COPD readmission within 30 days, n (%)***	12 (16)	7 (16.6)	5 (15.1)	0.65 (0.07, 5.6)	0.70
***COPD readmission within 90 days, n (%)***	20 (26.6)	12 (28.5)	8 (24.2)	0.82 (0.18, 3.6)	0.79
***Emergency department visit for COPD within 30 days, n (%)***	14 (18.6)	8 (19.0)	6 (18.1)	0.67 (0.13, 3.5)	0.64
***Emergency department visit for COPD within 90 days, n (%)***	22 (40.8)	11 (26.1)	11 (33.3)	1.01 (0.30, 3.4)	0.98
***All cause readmission within 30 days, n (%)***	16 (21.3)	11 (26.1)	5 (15.1)	0.36 (0.06, 2.3)	0.28
***All cause readmission within 90 days, n (%)***	28 (37.3)	17 (40.4)	11 (33.3)	0.71 (0.20, 2.5)	0.60

## Discussion

In this prospective study, we found that suboptimal PIFR is highly prevalent among hospitalized patients with AECOPD (i.e., more than half). Further, we found a linear relationship between HGS and PIFR. This relationship was robust and remained statistically significant after adjusting for potential confounders. We did not find a statistically significant association between PIFR and 30 or 90-day readmission.

Respiratory and non-respiratory skeletal muscle dysfunction has been previously described in COPD, which suggests biological plausibility for a relationship between PIFR and HGS.[29] The mechanisms of skeletal muscle dysfunction in COPD are still poorly understood, however it has been hypothesized that a net loss of muscle mass and myodysfunction due to intrinsic alterations such as mitochondrial abnormalities and loss of contractile proteins may occur, but also due to external factors such as hypoxia, hypercapnia and acidosis.[18,30] Chronic hypoxia interferes with protein synthesis in muscle cells, leading to muscle dysfunction.[31,32] Further, proinflammatory cytokines such as tumor necrosis factor- α levels are increased in COPD patients,[33,34] which can cause increased apoptosis of the skeletal muscle cells through a variety of mechanisms.[35,36]

In recent years, HGS use has been of great interest in different areas, including in the geriatric population, as a predictor of frailty and as a surrogate marker of cognitive function.[37] In a large metanalysis of 23,400 patients with cardiovascular disease, HGS emerged as an independent predictor of cardiac death, all-cause mortality and hospital admission.[38] Further, the clinical importance of HGS test in patients with COPD has also been reported in numerous studies.[39,40] HGS was found to be lower in patients with COPD as compared to healthy subjects.[15] Reduced HGS is associated with increased risk of hospital readmission due to acute exacerbation of COPD.[41] In one study, patients with COPD had a 15% decrease in maximum strength as compared to the control group.[42] In addition to muscle strength, in COPD patients HGS has been correlated well with overall exercise capacity, as measured by 6 minute walk test,[42–44] which has been found to be a good determinant of global quality of life and general mortality in these patients.[45–47] However, in spite of its importance, exercise capacity is not routinely measured in clinical practice because it entails time and space, and sometimes cannot be performed due to comorbid conditions such as arthritis, heart or cerebrovascular disease. As such, HGS has been recommended as a marker of muscle strength, especially when 6-minute walk test cannot be performed.[42,43]. Moreover, an association between HGS and forced expiratory volume in 1 second have been described in patients with COPD.[15,16]

The direct relationship between HGS and PIFR indicates that HGS may serve as an indirect tool to predict inspiratory muscle strength, as it can be performed at the bedside and is easily repeatable in hospitalized patients.[48] A formal measurement of inspiratory muscle strength requires special devices that are often only available in research settings, and hence not feasible for routine clinical purposes. Whether interventions aimed at increasing skeletal muscle strength also result in improvement of PIFR remains unknown, but such interventions are worth investigating.

As compared to patients in outpatient setting, we found in our study population a higher prevalence of S-PIFR. Mahler et al. found in 213 patients with stable COPD that 19% had S-PIFR (<60 L/min) against the resistance of Discus; S-PIFR was associated with age, female gender and lower percent predicted forced vital capacity and inspiratory capacity compared to O-PIFR.[10] However, the prevalence rate in our study is consistent with other studies conducted in inpatient setting.[13,19] For example, in a multicenter prospective study of 268 hospitalized patients with AECOPD, Sharma et al observed 81 patients with S-PIFR (32%); interestingly, the S-PIRF group was comprised mostly by older women.[19] In another retrospective study of hospitalized patients with AECOPD the prevalence of S-PIFR was 52% (64/123 subjects), and the majority were older than 65 years. The high prevalence rate may be due to the catabolic nature of the acute illness and coexistent metabolic derangements such as hypoxia, hypercapnia and acidosis, hence AECOPD leading to weaker respiratory muscles.

One may hypothesize that S-PIFR is associated with higher 30 or 90-day readmission in patients discharged on DPIs, however no large prospective study to date has evaluated the effect of S-PIFR on re-hospitalization. In a retrospective study, Loh et al found significantly lower number of days to readmission in discharged subjects with PIFR<60 L/min.[13] While we did not find a relationship between PIFR and 30 or 90-day readmission rates, the study was not powered for these outcomes.

This study has notable strengths and some potential limitations. In this prospective study we managed to follow patients for readmissions, including hospitalizations in other institutions within 90 days from the index admission, with very few dropouts, as opposed to other similar studies [19,13] that evaluated readmissions to the same healthcare facility. A potential limitation is that we measured PIFR against the simulated resistance of one DPI, a Discus® DPI. Discus DPI is commonly used and has lower internal resistance than others such as Turbuhaler^®^ and Handihaler^®^ DPIs. Because we found high prevalence of S-PIFR (~50%) against low resistance DPI, the prevalence of S-PIFR is likely even higher when patients are discharged on DPIs with higher internal resistance. Furthermore, we did not perform routine pulmonary function testing or 6 minute walk tests to determine in a standard fashion the severity of global functional impairment. Moreover, we could not confirm patients’ adherence to DPI usage after discharge, issue that may impact 30 and 90-day readmission rates. Although there was trend toward higher 30 and 90-day all-cause readmission and 30-day COPD readmission in individuals with S-PIFR, this was not statistically significant. A larger sample size may yield more comprehensive results that could support this hypothesis.

This is the first study to investigate the association between PIFR with HGS in hospitalized patients with AECOPD. The HGS can be used as a surrogate measurement to assess inspiratory muscle strength. Whether interventions aimed at increasing skeletal muscle strength also result in improvement in PIFR remains unclear and needs future study. However, both PIFR and HGS measurements are simple and likely beneficial in clinical practice. As such, we recommend PIFR and HGS to be measured routinely in clinical setting in order to assess this dimension of frailty, and potentially to determine the proper DPI type upon discharge. As such, subjects with S-PIFR may benefit from lower internal resistance DPIs or even long-acting controller medications in nebulized form.

## Supporting information

S1 Raw data (XLSX)

## Abbreviations

AECOPD: acute exacerbation of chronic obstructive pulmonary disease
PIFR: peak inspiratory flow rate
DPI: dry powder inhaler
Mmrc: modified Medical Research Council
CAT: COPD Assessment Test
HGS: hand grip strength
